# The Interaction Effect of Laser Irradiation and 6-Benzylaminopurine Improves the Chemical Composition and Biological Activities of Linseed (*Linum usitatissimum)* Sprouts

**DOI:** 10.3390/biology11101398

**Published:** 2022-09-25

**Authors:** Ahlem Zrig, Basma Najar, Shereen Magdy Korany, Abdelrahim H. A. Hassan, Emad A. Alsherif, Anis Ali Shah, Shah Fahad, Samy Selim, Hamada AbdElgawad

**Affiliations:** 1 Faculty of Sciences of Gabès-City Erriadh, Zrig, Gabes 6072, Tunisia; 2 Department of Agricultural, Food and Agri-Environmental Sciences, University of Pisa, Via del Borghetto, 80, 56124 Pisa, Italy; 3 Department of Biology, College of Science, Princess Nourah Bint Abdulrahman University, P.O. Box 84428, Riyadh 11671, Saudi Arabia; 4 Department of Food Safety and Technology, Faculty of Veterinary Medicine, Beni-Suef University, Beni-Suef 62521, Egypt; 5 Department of Botany and Microbiology, Faculty of Science, Beni-Suef University, Beni-Suef 62521, Egypt; 6 Biology Department, College of Science and Arts at Khulis, University of Jeddah, Jeddah 21959, Saudi Arabia; 7 Department of Botany, Division of Science and Technology, University of Education, Lahore 5477, Pakistan; 8 Hainan Key Laboratory for Sustainable Utilization of Tropical Bioresource, College of Tropical Crops, Hainan University, Haikou 570228, China; 9 Department of Clinical Laboratory Sciences, College of Applied Medical Sciences, Jouf University, Sakaka 72341, Saudi Arabia

**Keywords:** priming, laser light 6-benzylaminopurine, *Linum usitatissimum* sprouts, phytochemicals, microbial activities, antioxidant activity

## Abstract

**Simple Summary:**

Recently, novel food processing technologies have been used for the quality improvement of fruits and vegetables. Laser light application and 6-benzylaminopurine (BAP) priming are known to be promising approaches to improve the nutritional values of plants. In this study, we carried out novel food processing technologies using laser light and 6-benzylaminopurine (BAP) priming for the quality improvement of linseed sprouts. The combined treatments improved the accumulation of minerals, vitamins, and amino acids and enhanced the N-metabolism. Moreover, the application of both treatments combined enhanced the antioxidant capacity by increasing fatty acids, phenols, and flavonoids, as well as antimicrobial activity.

**Abstract:**

Even though laser light (LL) and 6-benzylaminopurine (BAP) priming are well-known as promising strategies for increasing the growth and nutritional value of several plants, no previous studies have investigated their synergistic effect. Herein, we investigated the effects of laser light, 6-benzylaminopurine (BAP) priming, and combined LL-BAP treatment on the nutritional value, chemical composition, and the biological activity of *Linum usitatissimum* sprouts. The fresh weight, leaf pigments, primary and secondary metabolites, enzymes, and antimicrobial activities were determined. A substantial enhancement was observed in the growth characteristics and leaf pigments of laser-irradiated and BAP-primed sprouts. Furthermore, the combined treatments improved the accumulation of minerals, vitamins, and amino acids, and also enhanced the N-metabolism more than LL or BAP alone. Furthermore, the combined priming boosted the antioxidant capacity by increasing the contents of fatty acids, phenols, and flavonoids. Antimicrobial activity and the highest increase in bioactive compounds were recorded in linseed sprouts simultaneously treated with LL and BAP. This work suggests that priming *L. usitatissimum* sprouts with laser light and BAP is a promising approach that can improve the nutritional value and health-promoting impacts of *L. usitatissimum* sprouts.

## 1. Introduction

Early plant seedlings known as sprouts have recently been used to improve the nutritional and functional characteristics of plants [1]. The quality and potential bioactivity of sprouts are mainly determined by the seed quality and conditions of germination [2]. For instance, sprouts have attracted a lot of attention because of the substantial amount of bioactive phytochemicals that they possess [3]. Moreover, sprouts are rich in essential amino acids, unsaturated fatty acids, minerals, antioxidants, and vitamins, which support their biological role as antioxidants and anticancer agents. Improving the phytochemical contents in many sprouts to boost their nutritional value has been well studied. To this end, several techniques have been applied to improve the yield and nutritional value of plants [4]. Novel food processing technologies have been used to assess the quality of various species [5]. Pulsed light could substitute current thermal processing technologies to restrict the further quality loss of a poor-quality raw material, especially when used for seed treatment. Laser light (He–Ne), used as low energy, is considered as an environmentally friendly method used to enhance plant growth [6]. Moreover, the bio-stimulating properties of laser light increase plant nutrition and productivity. In fact, several studies have shown that laser light can enhance the biological activities of sprouts [7]. Furthermore, laser light enhanced the biomass, mineral contents, and antioxidant metabolites of buckwheat sprouts, as well as their anti-inflammatory benefits [8]. A comparison between laser-treated seeds and untreated seeds of *Cymbopogon proximus* showed that treated ones produced bioactive chemicals which are beneficial for health. Furthermore, laser light improves the biochemical, physiological, and nutraceutical qualities of medicinal plants [7]. Another eco-friendly and economical technique was adopted to curtail the effects of climate change, such as seed priming [9]. Seed priming improves growth parameters, enhances bioactive component accumulation, and increases antioxidant and anti-diabetic activity [10,11]. For instance, hormonal seed priming is frequently used to improve germination and agricultural crops in difficult settings [5]. The priming of seeds with hormones such as 6-benzylaminopurine (BAP) helps to invigorate the seed and facilitate the processes of seed germination and seedling emergence. Moreover, BAP priming changes the biochemical and molecular processes [12,13]. Priming with BAP improves photosynthetic rate, membrane integrity, and ionic levels in plants, and also enhances chlorophyll (Chl) production and biomass accumulation. Moreover, BAP priming enhances the formation of secondary metabolites [13].

Plants are elicited in many ways to enhance phytochemical content and bioactivity. To generate favourable bio-stimulatory properties and highlight the synergism of different priming treatments, a combination of different physical or chemical treatments has been applied. The pre-soaking of seeds and the application of elicitation with plant hormones are sustainable methods used to enhance bioactive compound accumulation in broccoli and radish sprouts, thus improving their antioxidant activities [14]. Similarly, the probable synergistic effects of laser light application on treated *Pelargonium graveolens* and mycorrhizal enhanced mineral and essential oil content as well as antibacterial activity [15].

Linseed (*Linum usitatissimum* L.) has attracted a lot of attention in recent decades as a new source of high-value raw material for both food and industrial applications [16]. Linseed has been used for human nutrition since ancient times when it was employed not only for fibre production and various industries, such as paints, drying oils, and coatings, but also for medical and nutritional purposes [17]. Linseed seeds have become a very promising functional food due to the high number of nutrients and biologically active substances contained in them, such as phytoestrogenic lignans, phenolic compounds, high-quality proteins, and dietary fibres [18].

Considering these recent discoveries, we aimed to investigate the possible synergistic favourable effects of laser light treatment and 6-benzylaminopurine (BAP) priming on the levels of phytochemicals and bioactivity of linseed sprouts for the first time. In comparison to the effect of each elicitor independently, we expected that the combined elicitors might increase the bioactive content and enhance the antioxidant and biological activities of *L. usitatissimum* L.

## 2. Materials and Methods

### 2.1. Experimental Set-Up

*L. usitatissimum* seeds were obtained from Agricultural Research Centre, Giza, Egypt. Four groups were treated with different treatments: a control group, a laser-irradiated group using helium–neon (He-Ne, equipment whitening, laser II, DMC Equipment Ltd., São Carlos, SP, Brasil) for 5 min at 632 nm, 5 mW and 500 mJ energy. The beam diameter was 1 mm and the distance between the laser source and the seeds was 12 cm. The BAP (priming in 25 µM solution for 8 h) group and combined treatment (laser + BAP priming) were both studied.

Each group contained 100 seeds. Linseed seeds were germinated on trays containing vermiculite. Germinated seeds were watered (Milli-Q water) two times per week. Hoagland nutrient solution was supplied once at the start of the experiment to nourish the seedlings. All treated sprouts were grown in a climate-controlled chamber at 21/18 °C, over a 16/8 h day/night photoperiod (150 µmol PAR m^−^^2^ s^−^^1^, with 60% humidity). Sprouts from each tray that had been growing for ten days were weighed to measure the fresh weight and stored at −80 °C for a biochemical examination later. For each measurement, 15 plants from each tray were combined and utilized as biological duplicates. Three duplicates of each experiment were conducted.

### 2.2. Analysis of Leaf Pigments 

The leaf chlorophyll and carotenoid contents were determined, and then about 0.2 g of fresh leaves was ground in liquid nitrogen and homogenized for 30 s in 5 mL of 95.5% acetone. After homogenization, the extracted samples were centrifuged for 20 min (14,000× *g*, 4 °C). Then, the sample’s clear supernatant was filtered across 0.45 μm of Acrodisc GHP filter. Pigments were analysed with the reversed phase of HPLC (Shimadzu SIL10-ADvp). On a silica-based C18 column (Waters Spherisorb, 5 m ODS1, 4.6 250 mm, employed at a temperature of 40 °C), different types of pigments were separated. The mobile phase (A) contained 81:9:10 acetonitrile–methanol–water and solvent (B) contained 68:32 methanol–ethyl acetate. At room temperature, the mobile phase flowed at a rate of 1.0 mL/min. Pigments were then detected using a diode array detector at three wavelengths (647, 664, and 462 nm). Shimadzu Lab Solutions Lite software was used to measure pigments concentrations [19].

### 2.3. Analysis of Mineral Contents

For mineral contents analysis, 200 mg of dried linseed sprout was mineralized in HNO_3_/H_2_ O solution [20]. Both macro and micro minerals were determined by inductively coupled plasma mass spectrometry (ICP-MS, Bremen, Germany). A solution of nitric acid 1% was employed as the standard. The detection and quantification limits (LODs) were, respectively, 0.0002 and 0.01 g kg^−1^ and 0.004 and 0.3 g kg^−1^.

### 2.4. Analysis of Chemical Properties

#### 2.4.1. Sugar Quantification

Next, 100 mg of sprouts was extracted in acetonitrile–water (2 mL, 1:1, *v*/*v*) for 2 min [21]. The obtained extract was used to identify sugars with HPLC chromatography. For the mobile phase, a mixture of acetonitrile and HPLC grade water at a ratio of 75:25 (*v*/*v*) was used. The column temperature and injection volume were set at 30 °C and 20 μL, respectively. Sugar quantification was based on peak areas and a comparison with a calibration curve obtained with the corresponding standards ranged from 1 to 10 mg/100 mL of acetonitrile–water (1:1, *v*/*v*). Linseed sprouts were mixed with alpha-amylase (pH 6, 102 °C for 25 min) and extracted in ethanol to determine the fibres.

#### 2.4.2. The Quantitative Estimation of Alkaloids, Saponin, Steroids, and Tannin

To measure the alkaloid quantity in sprouts, samples were extracted in acetic acid–ethanol (20%, *v*/*v*). The filtration of the extract was precipitated with NH_3_ OH. The precipitate was collected, washed with diluted NH_3_ OH, and dried. After this, the residue was weighed. To quantify the saponin, sprouts were extracted in 20% ethanol [21]. At 60 °C for 4 h, the extraction mixture was heated. Then, 60 mL of n-butanol was added to the concentrate after the solvent had evaporated, and it was then washed with aqueous sodium chloride (5 percent, NaCl). The remaining solution was dried at 45 °C and the absorbance of the sample was measured at a wavelength of 527 nm. The total saponin content was expressed as 100 g^−1^ of oleanolic acid equivalents. To quantify the total steroid content, after being occasionally shaken and homogenized with acetone for 30 min in a water bath, 10 mg of the sample was kept on a rotary shaker for 24 h at 200 rpm. Sulfuric acid (4 N, 2 mL), iron (III) chloride (0.5 percent *w*/*v*), and potassium hexacyanoferrate (III) solution (0.5 percent *w*/*v*, 0.5 mL) were added to 1 mL of the test extract of the steroid solution. The mixture was incubated for 30 min in a water bath maintained at 70 °C with shaking before being diluted, and the absorbance was then measured at 780 nm [21]. To determine the total tannin, 0.05 g of fresh sprouts was extracted by Fe_2_ SO_4_, 95 mL of N-butanol, and 5 mL of HCl (35%). After filtration, 0.4 mL of aliquots was combined with 7.5% of Na_2_ CO_3_ and Folin–Ciocalteu reagent (1:10). The absorbance was read at 765 nm. Results were expressed as milligrams of gallic acid equivalent per gram of the sample dry weight using gallic acid as the standard.

#### 2.4.3. The Quantification of Total Lipid and Protein Contents

To quantify the total lipid content, linseed sprouts were extracted in a solution of chloroform–methanol (2:1, *v*/*v*). The pellets were re-dissolved in a solution of toluene–ethanol (4/1, *v*/*v*), after being centrifuged for 15 min at 3000× *g*. The extract was then combined with a saline solution. After the isolated lipids were concentrated, the amount of total lipid was determined. For total protein analysis, sprouts were extracted in NaOH (5 mL, 1 M) at 37 °C for 60 min. Folin–Lowry procedures were used to measure the concentrations, and the standards’ absorbance was measured at 660 nm in comparison to a blank [22]. 

### 2.5. The Determination of Amino Acids

To evaluate the amino acids, 200 mg of sprout was homogenized in ethanol (80 percent) and centrifuged at 22,000× *g* for 25 min [23]. The pellet was then passed through a Millipore filter with a pore size of 0.2 m. Amino acid concentrations were measured using the Waters Acquity UPLC-tqd system with a measurement at 254 nm, low pressure, and an acetonitrile–water mobile phase.

### 2.6. The Determination of Individual and Total Polyphenol and Flavonoid Contents

With 100 mg of sprouts, ethanol was used to extract the polyphenols and flavonoids to determine their total content (80 percent) [24]. For 20 min, the extract was centrifuged at 4 °C. The Folin–Ciocalteu assay was used to quantify the phenolic content, with gallic acid serving as the control. The total flavonoid content was estimated using the modified aluminium chloride colorimetric method, with quercetin as the standard. As previously stated by Gomaa and AbdElgawad, the HPLC-grade CH_3_ OH method was utilized to identify the specific polyphenols and flavonoids [22].

### 2.7. The Determination of Vitamin Contents 

Using HPLC techniques, the amounts of vitamin C, thiamine, riboflavin, and tocopherols in sprouts were measured [20]. Following this, 0.1 N of HCl was used to homogenize the fresh leaves in order to extract the thiamine and riboflavin. For riboflavin, the absorbance measurements were made at 453 nm and 580 nm. Moreover, the absorbance measurements of thiamine were made at 366 nm and 453 nm. By utilizing meta-phosphoric acid, ascorbate was extracted. Vitamin E was isolated and then quantified using HPLC on a Particil Pac 5 m column [20].

### 2.8. The Determination of Fatty Acid Compounds and Their Total Contents

Using the GC/MS (MSD 5975 mass spectrometer (Hewlett Packard, Palo Alto, CA, USA), the levels of fatty acids in plant samples were quantified [25]. The concentration of each molecule was calculated by comparing the peak area of each chemical to a calibration curve of the pertinent standard.

### 2.9. Analysis of N, Ammonium, and Nitrate Contents

The method proposed by Cataldo et al. [26] was used to determine the amount of nitrate. H_2_SO_4_ solution and salicylic acid were combined with the filtrate. To maintain the reaction’s pH at 12, 2 M NaOH was added. At 410 nm, the absorbance was measured. At 630 nm, the ammonium concentration was detected. By mineralizing the dried leaves in H_2_SO_4_-H_2_O_2_, the total nitrogen content was ascertained.

### 2.10. The Determination of Total Antioxidant Capacities

To estimate the total antioxidant capacity (TAC), fresh sprouts were mixed with ethyl acetate and phosphate buffer (50 mM) at pH 7.8 and centrifuged at 15,000 rpm for 15 min at 4 °C. The absorbance of each sample was measured at 532 nm. The oxygen radical absorbance was used in the ferric-reducing antioxidant power (FRAP) experiment to assess antioxidant capacity (ORAC). Then, 0.1 g linseed sprout was extracted in ethanol (80%) and centrifuged at 4 °C for 25 min at 14,000 rpm [27]. After incubation at room temperature, the absorbance was measured at 517 nm for FRAP. A mixture of linseed sprout extracts and 2,2-diphenyl-1-picrylhydrazyl (DPPH) or ORAC (in the presence of Cu^2+^ and H_2_O_2_) was incubated and detected at 517 nm. Phosphatidylcholine was sonicated for two hours on ice in a 10 mM phosphate buffer (pH 7.4) to produce liposomes for the anti-lipid peroxidation. At 532 nm, the absorbance of each sample was measured in comparison to the control.

### 2.11. Antibacterial Activity

Using the disc diffusion method, the studied sprouts’ antibacterial activity was assessed [15]. Using a vernier caliper to measure the inhibition zone, the antibacterial activity was ascertained. Using the disc diffusion method (a bacterial suspension of 106 CFU/mL of the bacterial test strain dispersed over Muller–Hinton agar), ethanol extracts of linseed sprouts were examined. The discs loaded with extracts (10 g/disc) and those loaded with ethanol served as positive and negative controls, respectively. These discs were put on agar plates with the tested bacteria and left to incubate for 24 h at 37° C. Vernier calipers were used to measure the inhibition zones.

### 2.12. Statistical Analyses

The R statistics program was used to conduct a statistical analysis (Gplot, Agricola). As a post-hoc test, one-way analysis (ANOVA) and Tukey’s test (P 0.05) were used. The principal component analysis procedure was carried out using the R statistics package. At least three instances (*n* = 3) were conducted. Using the Corrplot package, correlation analysis was performed on all the data.

## 3. Results

### 3.1. Treatment Effect on Biological and Biometric Characteristics

Depending on the treatment, large variations in the biometric and pigment content of sprouts were observed (Table 1). When used separately, laser light and BAP priming each have no significant effect on the fresh weight of sprouts, Chla, as well as their carotenoids and total chlorophyll content. On the contrary, compared with the control, a significant difference was observed between each laser and BAP treatment for DW and BAP for the amount of chlorophyll b (*p* < 0.05). The highest pigment content, as well as FW and DW, was obtained by combined treatments. Except for Chla, which was unaffected, all of the measured parameters showed statistically significant variations (*p <* 0.05) when compared to the control. The percentage of enhancement ranged from 16% in the total Chl to 80% for the FW.

### 3.2. Primary Metabolites

**Figure 1:**
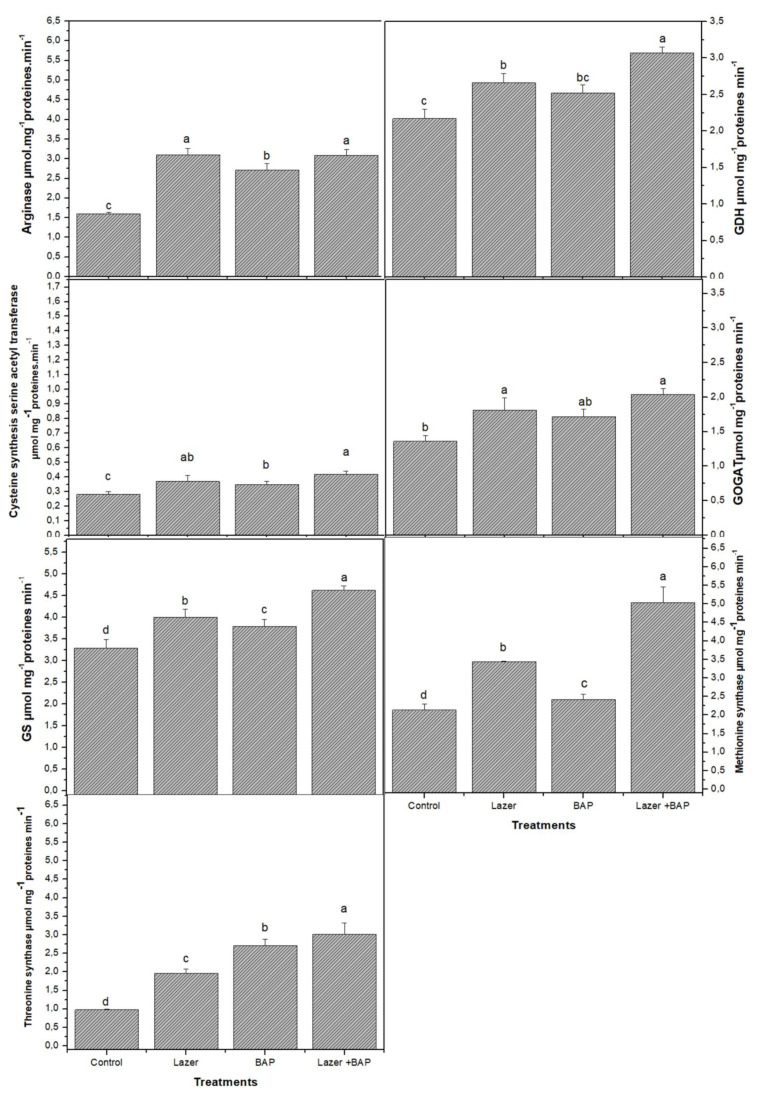
The effect of laser irradiation, BAP, and laser irradiation with BAP priming on the amino acid metabolism of linseed sprouts. GS: glutamine synthase, GDH: glutamine dehydrogenase, and GOGAT: glutamate synthase. The values represent the mean of three replicates ± standard error. The small letters (a–d) above the means show the differences (*p* < 0.05) between the treatments by Tukey’s test.

Plant growth and vital life functions rely on primary metabolites in order to function properly. Twenty amino acids were detected in linseed sprouts. The highest values were recorded for glutamine and glutamic acid (46.5 mg·g^−1^ FW and 34.0 mg·g^−1^ FW, respectively) (Table 2). Even though the difference between the two treatments was statistically minor, the laser light effect increased their biosynthesis rather than BAP priming (*p* > 0.05). Overall, the laser light boosted the biosynthesis of almost all the studied AAs, while the BAP seemed to be less effective on them, as only 9 out of 20 AAs showed significant differences compared to the control (*p* < 0.05). Combined treatment actively induced the synthesis of AAs. Interestingly, therein the greatest quantities of glutamine and glutamic acid were observed (62.53 and 50.90 mg·g^−1^ FW, respectively), with an increase of 34.4 and 49.7%, respectively, compared to the control. Histidine, the third major AA produced by the control group, was negatively affected by both laser light and BAP treatments as its quantity decreased; even the combined treatment could not affect its value. Furthermore, the laser light and BAP priming enhanced the activity of enzymes involved in the biosynthesis of AA and N metabolism (Figure 1). For instance, the highest activity of glutamine synthase (GS), glutamine dehydrogenase (GDH), and glutamate synthase (GOGAT) was recorded in sprouts treated with both laser light and BAP priming. The same increase trends were observed in arginase, thymine synthetase, and methionine synthetase.

The same trend was also observed in vitamins where the combined treatment significantly increased their values (*p* < 0.05). Vitamins revealed sensitivity to both BAP and laser light treatments, although vitamin E (especially β and γ-tocopherol) showed an insignificant slight decrease in their values subsequent to laser light exposure. The quantity of alpha-tocopherol, the main vitamin produced by the control germs, was increased by all the treatments; however, the combined one showed the strongest effect.

Fatty acids, another primary metabolite, behaved differently upon exposure to the three treatments. In comparison to the BAP, the laser light and combined treatment resulted in a minor increase in hexadecanoic acid (16:0) and octadecanoic acid (18:0), with their values revealing a statistically significant difference compared both to the control and the cited treatments. Additionally, octadecenoic acid (18:1) was especially fostered by combined treatment and its quantity rose from 30.86 mg·g^−1^ FW to more than 52 mg·g^−1^ FW. Interestingly instead, the three treatments elicited an increase in octadecanoid acid (18:2) in the same manner. Furthermore, laser light, alone or combined with BAP, has nearly doubled the total protein amount, as compared to the control and BAP alone treatment. A doubling of the content was also observed in sugars, but only when merging laser light to BAP. A reduction in the crude fibre value was noticed in all analyses

### 3.3. Secondary Metabolites

Implied in defence mechanisms, the biosynthesis of secondary metabolites was influenced by the treatments in different ways. In comparison with the untreated control, significant improvements in the alkaloid yield (1.34- and 1.78-fold increases) were obtained when the sprouts were sprayed with only BAP or if they were unified with the emission of laser light (Table 3). The same tendency was also underlined in saponin production. Tannin content initially followed a linear increase and then underwent an insignificant decline. A drastic drop in caffecic acid production when BAP was applied (about a 40% reduction). Moreover, a 25% reduction was observed in the total fenol, even though the laser light hither was responsible for this subsidence. Except for kanferol, the biosynthesis of all other flavonoids was disheartened by the light. Overall, both polyphenols and flavonoids were incited by the combination between BAP and laser light.

Ash indicates the mineral matter content in plants and is the inorganic matter that remained after water and organic substances were removed by heating (Table 4). The minimum total ash recorded was 1.6 ± 0.47% in control sprouts, while the maximum total ash recorded was 3.2 ± 0.25% in those grown under laser treatment combined with BAP.

The main nutrients for plant growth were nitrogen, phosphorus, and potassium (N, P, and K, respectively). Their output varied depending on the treatment. Compared to the control, laser treatment combined with BAP evidenced the greatest value of all assessed minerals. Separately, instead, the laser pointed out to be better than BAP on mineral amounts, except for azote N (40.90 mg/g in sprouts exposed to laser vs. 46.02 mg/g in primping with BAP) and sodium (Na) (0.25 vs. 0.79 mg/g, respectively), while an insignificant effect was observed on P (Table 4). 

### 3.4. Biological Activity

The effects of laser light, BAP, as well as combined treatments were tested for their antimicrobial activity (Table 5). Antibacterial activity was tested against both Gram-positive and Gram-negative microorganisms. Laser light evidenced the best effect on almost all Gram-positive bacteria and >50% of those belonging to Gram-negative ones. An opposite reaction was noted for the laser + BAP treatment effect on *Enterococcus faecalis*, *Proteus vulgaris*, and *Enterobacter aerogenes*. While both laser treatment and BAP treatment separately induced a decrease in the efficacy of sprouts on them, there was an increase of about 73% in sprouts exposed to the combined treatment against *Salmonella Typhimurium*, which seemed to be the most sensitive one to this treatment. Whereas. *Streptococcus salivarius* was the most sensitive bacteria to the effect of sprouts. Concerning the antifungal activity, the study focused on two yeast types belonging to the candida genus and *Aspargellus flavus* fungi of preharvest and postharvest seed crops. When compared to the control group, all treatments improved the flax seed antifungal activity. The laser treatment bestowed the greatest antifungal activity out of all tested fungi. 

**Figure 2:**
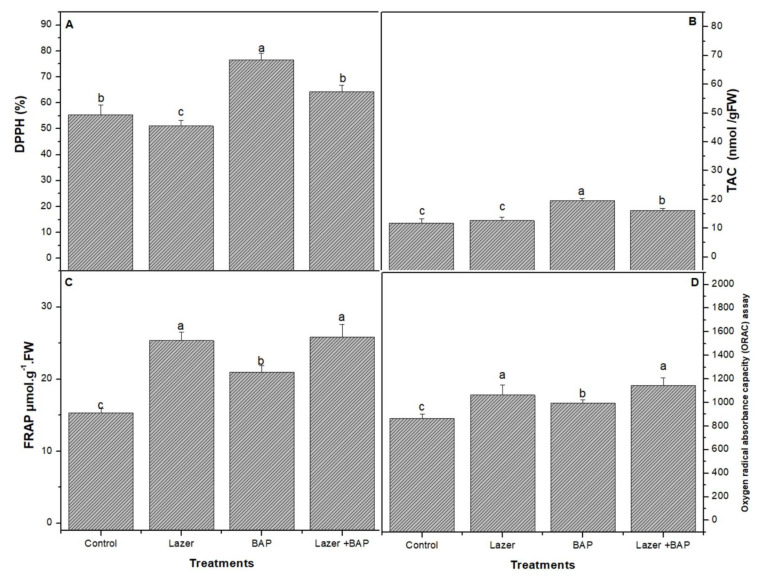
The effect of laser light, BAP, and laser with BAP priming on DPPH (%) (**A**), TAC (nmol/gFW) (**B**), and FRAP µmol.g^−1^.FW (**C**) and oxygen radical absorbance capacity (ORAC) (**D**) assay of linseed sprouts. The values represent the mean of three replicates ± standard error. The small letters (a, b and c) above the means show the differences (*p* < 0.05) between the treatments using Tukey’s test.

The antioxidant activity of linseeds was compared between the three treatments using three spectrometric methods: DPPH, FAR, and ORAC assays. BAP enhanced the DPPH activity (Figure 2A) and was assessed by FRAP (Figure 2B).

**Figure 3:**
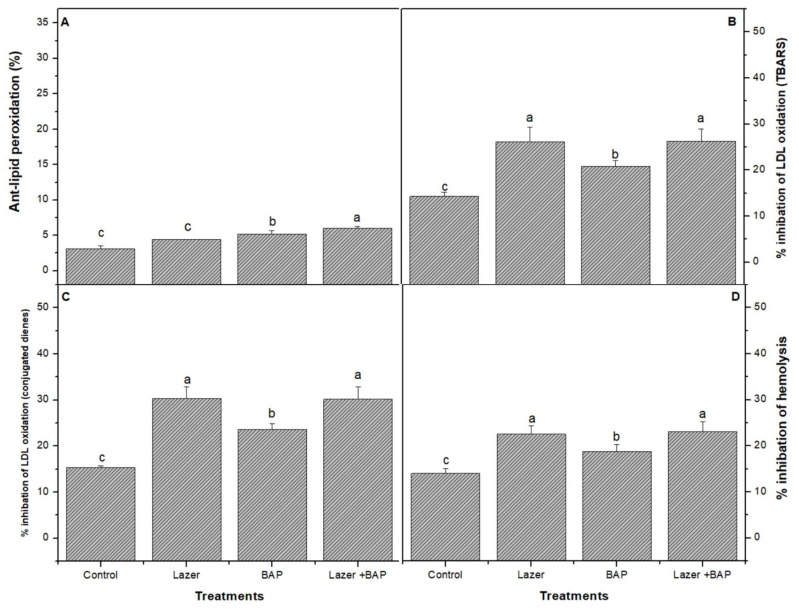
The effect of laser light, BAP, and laser with BAP priming on the % inhibition of LDL oxidation (**A**), the % anti-lipid peroxidation TBARS (**B**), the % inhibition of LDL oxidation (conjugated dienes) (**C**), and the % inhibition of the haemolysis of linseed sprouts (**D**). The values represent the mean of three replicates ± standard error. The small letters (a–d) above the means show the differences (*p* < 0.05) between the treatments using Tukey’s test.

The combined treatment presented a higher activity of the ORAC (Figure 2) radical scavenging than the other treatments. The combined one induced a significant increase (*p* < 0.05) in the lipid peroxidase product (Figure 3), including thiobarbituric-acid-reactive substances (TBARS) (e.g., malondialdehyde), when compared with the control sample.

### 3.5. Principal Component Analysis (PCA) and Correlation Analysis

**Figure 4:**
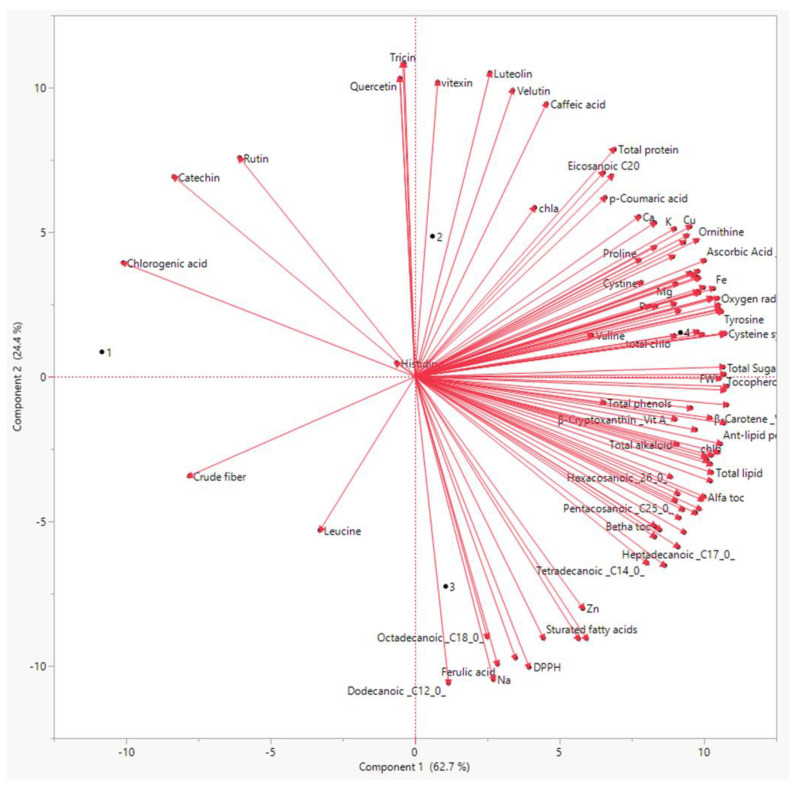
Principal component analysis of all measured physiological and biochemical parameters of the linseed sprouts. 1: Control, 2: laser, 3: BAP, and 4: L + BAP.

For all of the obtained results, principal component analysis (PCA) was used to summarize the differences between the three priming methods on linseed sprouts (Figure 4).

**Figure 5:**
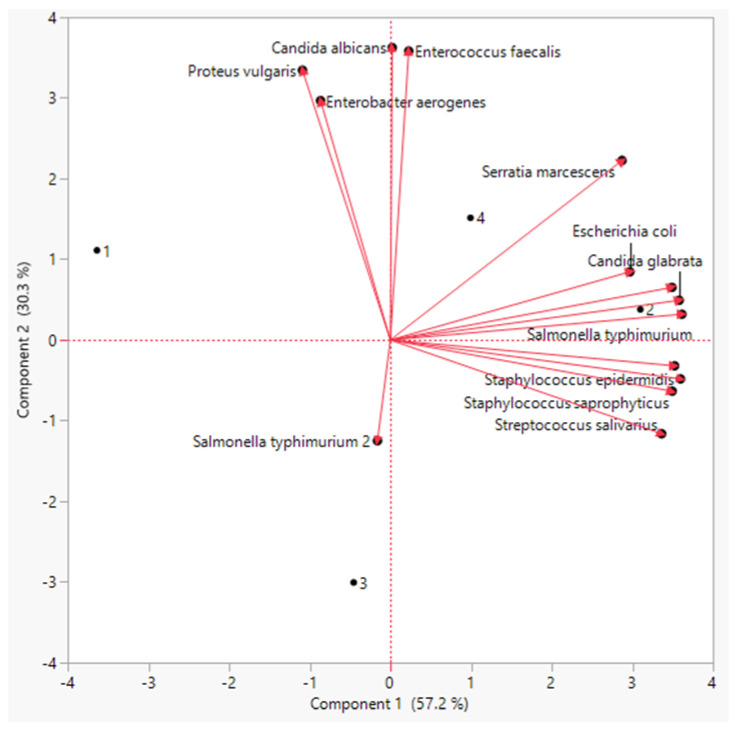
Principal component analysis of the antimicrobial activity of the linseed sprouts.

The PCA showed that there was a distinct treatment based on the bioactive chemicals. The PCA revealed that these metabolites differed by two primary components between the three treatments (component 1: 71% and component: 2 20%). Polyphenols, flavonoids, antioxidants, and amino acids were the most prominent PCA1 metabolites in sprouts individually treated with both laser and BAP priming. Similarly, the PCA revealed a distinct difference in antimicrobial activity between the regimens (Figure 5). The PCA2 scored well in antibacterial activity metrics, particularly in the laser +BAP cluster (LP).

**Figure 6:**
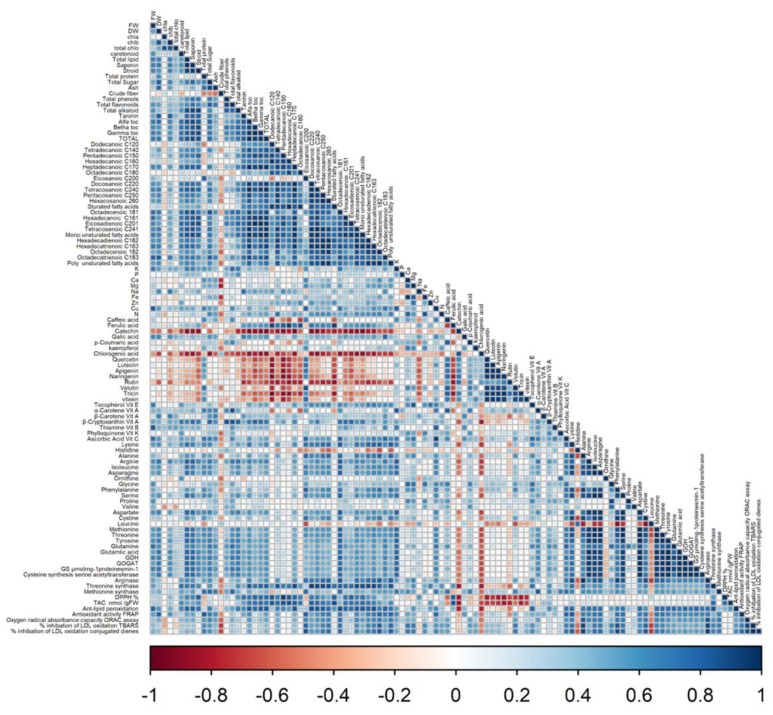
The cluster correlation of primary and secondary metabolites of Linseed sprouts treated by laser light, BAP, and laser light + BAP priming treatments. The graph’s horizontal axis shows different treatments for each species, and the vertical axis shows different phytocompounds, amino acids, and nitrogen content. Colour gradients represent the different values of contents under KNO3 priming compared with that of the control.

According to Pearson’s correlation coefficients between biological and chemical factors (Figure 6), the biomass accumulation of linseed sprouts was strongly correlated by all secondary metabolic, such as total phenols and flavonoids, as well as the antioxidant enzymes. Vitamins, amino acids, and enzymes involved in N metabolism were all favourably associated with total fresh and dry weights.

## 4. Discussion

Laser bio-stimulation is a result of the seeds’ ability to absorb and retain light energy, convert it to energy kept in chemical compounds, and then use it in seed germination and plant growth. Indeed, the outcomes of this investigation indicate that laser light improved the growth parameters of the studied linseed sprouts. This agrees with previous studies which confirmed the enhancing effect of laser light on the great development of linseeds [28]. Furthermore, many studies have previously shown that laser light has a strong influence on the biomass accumulation of several sampling, such as faba bean [29], maize [30], and radish [31], which is consistent with our findings. Likewise, BAP priming led to an improvement in the growth parameters of linseed sprouts. According to Mangena [32], the application of BAP increase the growth parameters of soybean sprouts. This report, together with that of Chaudhuri [33], emphasized the role of BAP in increasing the plant biomass. Interestingly, the combined physical and chemical priming seemed to enhance the growth parameters in linseed sprouts and reach their highest level, as compared to control and both laser light and BAP treatment separately. Nevertheless, as clearly demonstrated by the results in this study, the methods of pre-sowing priming seeds indicate that the potential role of laser light in seeds could increase the positive influence of the BAP pre-sowing treatment. Apparently, laser light enhanced the potential role of BAP in breaking seed dormancy and improved germination as a consequence of increasing the growth parameters. Indeed, the laser light improves the energetic potential of phytohormone, which leads to the activation and stimulation of their biochemical metabolism. The increase in bio-energy levels in organisms can be related to the bio-stimulation effect of laser light on development and sprout growth. Furthermore, BAP has been proposed as a “first determinant” in the breaking of dormancy. In this regard, Vasilevski (2003) [34] demonstrated that BAP could cause tuber sprouting, but that this was dependent on the tissue sensitivity. Moreover, the increment of growth parameters recorded in linseed sprouts treated with laser light and primed with BAP appeared to be related to the increase in leaf pigments. We suggest that the increase in Chlb and carotenoid contents is due to an increase in the quantity of free radicals in seeds exposed to laser light application, which have a direct impact on seed metabolism [35]. In a previous report, it was demonstrated that the high concentration of BAP alone decreased Chlb and carotenoid content [36]. On the contrary, the increase in Chl b and carotenoid content seemed to be related to the effect of laser light. It is worth noting that the presence of BAP combined with laser light led to the promotion of membrane stability, and helped to maintain ionic levels, which then increase photosynthetic rates and improve growth parameters [5]. The positive synergic effect of laser light and BAP priming on linseed sprouts could be explained by the produced sugars (glucose), combined with O_2_ to produce cellular energy, C skeleton, and energy required for the bioactive metabolite’s biosynthesis by dark respiration. Moreover, our results demonstrated the enhancement of total proteins, lipids, sugars, and ash (primary metabolites), as well as phenolics, flavonoids, saponins, steroids, and alkaloids (secondary metabolites), which was observed in sprouts treated with laser light and BAP priming separately, but the greatest improvement was recorded in the laser–BAP priming linseed sprouts. In previous studies, the laser light and BAP priming increased the primary and second metabolites [7]. Interestingly, priming with physical and nonphysical treatments was more efficient in enhancing both the primary and secondary metabolites. 

Mineral deficiencies have been related to poor human health outcomes. Sprouts have long been regarded to be rich in accessible minerals. Linseed plants have been cultivated for their nutritional value, as they are rich in minerals, especially P, Mg, and Ca, and contain very little salt [37]. Moreover, the high mineral element content of flaxseed sprouts, as a response to physical application or BAP priming, may thus improve the nutritional value of flaxseed sprouts. In this investigation, an enhanced mineral content was detected in laser–BAP-primed sprouts. A link between light perception and nutrient intake has been established in a number of studies [38]. The application of laser light, particularly, has been shown to boost the nutritional value of a variety of species [39]. According to our results, the combination of laser application and BAP priming in this study recorded a substantial synergism in the minerals’ uptake by linseed sprouts. This variation in mineral nutrient levels influences the specific amino acid composition of sprouts. In this study, the linseed primed with laser light and BAP, either alone or combined, displayed the highest level of all amino acids. The inability of human systems to manufacture specific amino acids has sparked interest in enhancing these necessary amino acid quantities in plants [40]. In this sense, the biosynthesis of essential amino acids, in addition to being involved in protein synthesis, is a crucial component that influences the plant’s nutritional value. Furthermore, amino acids are being employed to improve plant productivity and are regarded as natural plant growth stimulators [41]. Thus, the high content of amino acid levels found in laser treatment could be considered a key benefit of adopting this technology. In linseed sprouts, this increase in many amino acids in response to the priming with laser light and BAP has resulted in increases in total N and total protein [42]. Changes in amino acid levels could be explained by the increased activity of key enzymes involved in N metabolisms, such as GDH, GOGAT, and GS. Furthermore, the increase in cysteine, methionine, arginine, and threonine could be explained by the intensification in enzyme activity caused by the acceleration of seed germination cellular events. The increased metabolic activity of sprouts is caused by the seeds absorbing more energy from the laser light. Seeds irradiated with laser light promoted ATP synthesis, resulting in an increase in the crop bulk. Vitamin levels in linseed sprouts were higher in response to laser light and BAP priming in the current investigation. Previous research has shown that laser light as a physical treatment and BAP priming individually improve the vitamin content of several plants, including pea and lupine seeds [43]. Surprisingly, the combined effect of both priming treatments significantly enhanced all vitamins in this investigation. Clearly, the high concentration of BAP during priming boosted the activity of phytohormone in seeds, which stimulated the metabolic activity of linseed sprouts.

Linseed has gained a reputation as a promising functional food due to the high concentration of minerals and physiologically active substances, such as phenolic and flavonoids, found in its seeds. Phenolic chemicals are significant food metabolites that can help to prevent the onset of a variety of diseases, including cardiovascular and neurological diseases, as well as cancer [17]. These effects are due to phenolic compounds’ anti-inflammatory, anti-oxidative, and anti-carcinogenic properties, as well as their ability to control important enzyme functions. The effect of seed application with laser and BAP treatments, either independently or in combination, on the profiles of polyphenols and flavonoids of sprouts of linseed was examined. In linseed treatment, laser light therapy alone resulted in different degrees of improvement in phenolic levels, with improvements in gallic acid being particularly notable. Likewise, BAP priming alone showed the same increase. Individual phenolic and flavonoid results were consistent with total phenolic and flavonoid results. The obtained results are in line with previous research that demonstrated that laser treatment improved the phenolic and flavonoid profiles of plants like sunflower seeds [44] and soybean sprouts [45]. Meanwhile, preceding works have illustrated the effect of BAP priming on raising the phytochemical productivity such as phenolics and flavonoids [32,46]. Interestingly, the linseed sprouts primed with laser light and BAP, simultaneously, showed the highest levels of total phenolic and flavonoid contents. This is related to the synergistic action of BAP and laser light when used simultaneously. Many studies have shown that BAP priming can increase the production of phenolics and flavonoids [5]. Subsequently, the laser light pre-treatment seemed to boost the phenol and flavonoid contents. This could be due to the increased energy source of seeds, which is converted to chemical energy, which speeds up critical processes and enhances secondary metabolite production [47]. 

In this study, different methodologies were utilized to assess the antioxidant activity of linseed. Linseed plants’ antioxidant capabilities are mostly linked to active chemicals found in their tissues, such as phenolic and flavonoid molecules. However, because it is a complex mixture of many molecules, this could be owing to the large percentage of primary constituents, as well as the synergistic and antagonistic effects between these main elements and other constituents present in minor amounts. It is worth noting that all priming seed treatments enhanced the antioxidant activity in linseed sprouts, and the higher activity was recorded by the laser light and BAP priming. In line with our findings, the application of laser light and BAP enhanced the total antioxidant activities in buckwheat [48] and soybean sprouts [32]. Linseed sprouts were discovered to have a high overall antioxidant activity and were favourably linked with an increase in polyphenolic levels.

Using the disc diffusion method, the antibacterial potentials of *L. usitatissimum* sprouts were assessed following exposure to laser light and BAP priming in terms of the sizes of bacterial growth inhibition zones. Linseed sprout extracts were tested for antibacterial activity against foodborne bacterial pathogens, including *S. saprophyticus*, *S. epidermidis E. coli, P. aeruginosa, P. vulgaris, E. aerogenes, and S. Typhimurium*. Previous reports revealed that *L. usitatissimum* displayed high potential antimicrobial activity against different pathogens [49]. In this study, the pre-treatment with laser light and BAP separately enhanced the antimicrobial activity in linseed, but the highest increment was detected in sprouts treated with a combination of laser light and BAP. An increase in the levels of bioactive metabolites, such as phenolic and fatty content in linseed sprouts in response to laser light and BAP priming, could be attributed to an improvement in the antibacterial characteristics of the sprouts. A previous study pointed out the role of the phenolic compounds of *L. usitatissimum* in stimulating the degradation of bacterial DNA, as well as denying the gyrase activity. Linoleic acid preferentially inhibits a key component of bacterial fatty acid production, known as enoyl-acyl carrier protein (FabI) [50]. Oleic acid, another major unsaturated fatty acid, also exhibited the inhibition of FabI [50]. An interaction between vitamin content, especially vitamin C and D, and antibacterial activity was also reported [51]. It is worth noting that the presence of phenolic compounds, as well as fatty acids and lignans, is critical for *L. usitatissimum’s* antibacterial activity. 

## 5. Conclusions

Laser light and BAP alone improved the physiological and biochemical pathways involved in the formation of bioactive component, and also identified the targets for enhancing compound production in linseed sprouts. In addition, an increase in the levels of bioactive metabolites, such as phenolic and fatty content in linseed sprouts, in response to laser light and BAP priming, could be attributed to an improvement in the antibacterial characteristics of sprouts. Generally speaking, the synergism of laser light treatment and BAP priming of plants could be considered a promising tool to improve the nutraceutical and biological values of edible plants.

## Figures and Tables

**Table 1:** The effect of laser light treatment (5 nm at 632 nm), BAP priming (25 µM for 8 h), and laser combined with BAP treatment on the biometric characteristics and pigment contents of *Linum usitatissimum* sprouts.

	Control	Laser	BAP	Laser + BAP
FW g/plants	1.16 ± 0.19 ^b^	1.51 ± 0.19 ^ab^	1.60 ± 0.19 ^ab^	2.09 ± 0.10 ^a^
DW g/plants	0.34 ± 0.03 ^b^	0.43 ± 0.05 ^a^	0.44 ± 0.03 ^a^	0.49 ± 0.02 ^a^
Chlorophyll a (mg·g^−1^ FW)	3.46 ± 0.28 ^ab^	3.37 ± 0.42 ^b^	3.21 ± 0.11 ^b^	3.72 ± 0.14 ^a^
Chlorophyll b (mg·g^−1^ FW)	1.87 ± 0.05 ^b^	2.06 ± 0.34 ^ab^	2.27 ± 0.11 ^a^	2.47 ± 0.15 ^a^
Total Chlorophyll (mg·g^−1^ FW)	5.34 ± 0.23 ^b^	5.43 ± 0.72 ^b^	5.47 ± 0.14 ^ab^	6.20 ± 0.26 ^a^
Catenoids (mg·g^−1^ FW)	0.41 ± 0.04 ^b^	0.44 ± 0.01 ^ab^	0.49 ± 0.02 ^ab^	0.55 ± 0.01 ^a^

The superscript uppercase letters (a–d) indicate statistically significant differences between the samples. The statistical significance of the relative abundances was determined by Tukey’s post-hoc test, with *p* ≤ 0.05.

**Table 2:** The effect of laser light treatment (5 mn at 632 nm), BAP priming (25 µM for 8 h), and laser combined with BAP treatment on the primary metabolite biosynthesis response of *Linum usitatissimum* sprouts.

		Control	Laser	BAP	Laser + BAP
Amino Acids (mg·g^−1^ FW)	Lysine	1.4 ± 0.08 ^b^	2.63 ± 0.17 ^a^	2.33 ± 0.20 ^a^	2.67 ± 0.05 ^a^
	Histidine	1.7 ± 0.08 ^a^	1.13 ± 0.01 ^b^	1.29 ± 0.10 ^ab^	1.71 ± 0.08 ^a^
	Alanine	0.5 ± 0.02 ^b^	1.22 ± 0.11 ^a^	0.97 ± 0.17 ^b^	1.12 ± 0.11 ^a^
	Arginine	1.0 ± 0.02 ^b^	1.17 ± 0.13 ^b^	1.14 ± 0.08 ^b^	1.37 ± 0.03 ^a^
	Isoleucine	0.1 ± 0.01 ^b^	0.12 ± 0.01 ^a^	0.10 ± 0.02 ^b^	0.13 ± 0.01 ^a^
	Asparagine	0.5 ± 0.06 ^b^	0.90 ± 0.10 ^a^	0.75 ± 0.12 ^ab^	0.90 ± 0.05 ^a^
	Ornithine	0.1 ± 0.03 ^b^	0.12 ± 0.02 ^a^	0.10 ± 0.00 ^b^	0.13 ± 0.02 ^a^
	Glycine	0.6 ± 0.06 ^b^	0.70 ± 0.09 ^a^	0.62 ± 0.02 ^b^	0.80 ± 0.02 ^a^
	Phenylalanine	0.2 ± 0.02 ^c^	0.32 ± 0.00 ^a^	0.24 ± 0.04 ^b^	0.30 ± 0.02 ^a^
	Serine	0.2 ± 0.02 ^c^	0.29 ± 0.03 ^a^	0.24 ± 0.04 ^b^	0.30 ± 0.02 ^a^
	Proline	0.6 ± 0.06 ^b^	0.59 ± 0.07 ^b^	0.56 ± 0.03 ^b^	0.72 ± 0.01 ^a^
	Valine	0.2 ± 0.03 ^b^	0.22 ± 0.04 ^b^	0.23 ± 0.01 ^b^	0.29 ± 0.01 ^a^
	Aspartate	0.01 ± 0.00 ^b^	0.02 ± 0.00 ^a^	0.02 ± 0.00 ^a^	0.02 ± 0.00 ^a^
	Cystine	0.01 ± 0.00 ^b^	0.09 ± 0.01 ^a^	0.05 ± 0.03 ^ab^	0.07 ± 0.01 ^a^
	Leucine	0.1 ± 0.02 ^b^	0.01 ± 0.00 ^c^	0.10 ± 0.05 ^b^	0.11 ± 0.02 ^a^
	Methionine	0.008 ± 0.00 ^b^	0.01 ± 0.00 ^a^	0.01 ± 0.00 ^a^	0.01 ± 0.00 ^a^
	Threonine	0.05 ± 0.01 ^b^	0.07 ± 0.01 ^ab^	0.07 ± 0.01 ^ab^	0.08 ± 0.00 ^a^
	Tyrosine	0.5 ± 0.05 ^c^	0.65 ± 0.07 ^a^	0.60 ± 0.06 ^ab^	0.73 ± 0.04 ^a^
	Glutamine	46.5 ± 4.9 ^c^	53.32 ± 0.49 ^b^	50.3 ± 2.3 ^b^	62.53 ± 1.36 ^a^
	Glutamic acid	34.0 ± 3.63 ^c^	45.19 ± 7.7 ^ab^	43.00 ± 4.84 ^b^	50.90 ± 3.56 ^a^
Vitamins (mg·g^−1^ FW)	α-Carotene (Vit A)	0.4 ± 0.05 ^c^	0.52 ± 0.08 ^b^	0.23 ± 0.01 ^c^	0.82 ± 0.12 ^a^
	β-Carotene (Vit A)	0.1 ± 0.04 ^b^	0.16 ± 0.04 ^b^	0.20 ± 0.06 ^ab^	0.26 ± 0.06 ^a^
	β-Cryptoxanthin (Vit A)	0.06 ± 0.01 ^c^	0.06 ± 0.03 ^c^	0.11 ± 0.01 ^b^	0.19 ± 0.03 ^a^
	Thiamine (Vit B)	0.05 ± 0.01 ^b^	0.07 ± 0.02 ^b^	0.06 ± 0.02 ^b^	0.16 ± 0.08 ^a^
	Ascorbic acid (Vit C)	1.30 ± 0.03 ^b^	1.96 ± 0.06 ^a^	1.60 ± 0.18 ^b^	2.40 ± 0.15 ^a^
	Phylloquinone (Vit K)	0.12 ± 0.03 ^b^	0.14 ± 0.04 ^ab^	0.11 ± 0.01 ^b^	0.23 ± 0.05 ^a^
	α-Tocopherol (Vit E)	187 ± 84 ^b^	241.8 ± 10.5 ^ab^	302.56 ± 6.03 ^a^	323.16 ± 7 ^a^
	β-Tocopherol (Vit E)	74.8 ± 0.23 ^b^	73.0 ± 1.8 ^b^	96.66 ± 0.48 ^a^	105.81 ± 4.4 ^a^
	γ- Tocopherol (Vit E)	15.00 ± 0.17 ^b^	15.96 ± 0.02 ^b^	20.5 ± 0.15 ^ab^	22.2 ± 0.84 ^a^
	Tocopherol (Vit E)	0.5 ± 0.08 ^c^	0.91 ± 0.18 ^a^	0.89 ± 0.19 ^b^	1.07 ± 0.04 ^a^
Fatty Acids (mg·g^−1^ FW)	Dodecanoic acid (C12:0)	0.18 ± 0.01 ^b^	0.18 ± 0.00 ^b^	0.23 ± 0.00 ^a^	0.19 ± 0.00 ^b^
	Tetradecanoic acid (C14:0)	0.16 ± 0.00 ^b^	0.2 ± 0.00 ^b^	0.24 ± 0.02 ^a^	0.21 ± 0.01 ^a^
	Pentadecanoic acid (C15:0)	0.02 ± 0.00 ^ab^	0.02 ± 0.00 ^ab^	0.03 ± 0.00 ^a^	0.03 ± 0.00 ^a^
	Hexadecanoic acid (C16:0)	10.02 ± 0.12 ^b^	10.54 ± 0.01 ^b^	13.7 ± 0.10 ^a^	10.98 ± 0.05 ^b^
	Heptadecanoic acid (C17:0)	0.03 ± 0.00 ^b^	0.03 ± 0.00 ^b^	0.04 ± 0.00 ^a^	0.04 ± 0.00 ^a^
	Octadecanoic acid (C18:0)	9.84 ± 0.23 ^c^	10.29 ± 0.31 ^b^	11.45 ± 0.32 ^a^	10.07 ± 0.29 ^c^
	Eicosanoic acid(C20:0)	1.11 ± 0.04 ^b^	1.40 ± 0.04 ^a^	1.16 ± 0.01 ^b^	1.29 ± 0.04 ^a^
	Docosanoic acid (C22:0)	1.16 ± 0.01 ^b^	1.23 ± 0.01 ^b^	1.31 ± 0.02 ^a^	1.30 ± 0.03 ^a^
	Tetracosanoic acid (C24:0)	1.48 ± 0.03 ^c^	1.67 ± 0.01 ^b^	1.87 ± 0.03 ^a^	1.90 ± 0.04 ^a^
	Pentacosanoic acid (C25:0)	0.09 ± 0.00 ^c^	0.12 ± 0.00 ^b^	0.14 ± 0.00 ^a^	0.13 ± 0.00 ^ab^
	Hexacosanoic acid (26:0)	0.02 ± 0.00 ^b^	0.02 ± 0.00 ^b^	0.03 ± 0.00 ^a^	0.02 ± 0.00 ^b^
	Octadecenoic acid (18:1)	30.86 ± 0.60 ^c^	41.51 ± 2.31 ^b^	43.17 ± 2.39 ^b^	52.61 ± 2.29 ^a^
	Hexadecanoic acid (C16:1)	0.42 ± 0.01 ^a^	0.43 ± 0.02 ^a^	0.5 ± 0.01 ^a^	0.50 ± 0.01 ^a^
	Eicosadienoic acid (C20:1)	0.22 ± 0.00 ^b^	0.22 ± 0.01 ^b^	0.29 ± 0.01 ^a^	0.32 ± 0.01 ^a^
	Tetracosenoic acid (C24:1)	0.29 ± 0.01 ^b^	0.42 ± 0.03 ^ab^	0.51 ± 0.03 ^a^	0.52 ± 0.01 ^a^
	Hexadecatrienoic acid (C16:2)	0.90 ± 0.01 ^b^	1.22 ± 0.03 ^b^	1.35 ± 0.01 ^a^	1.28 ± 0.04 ^ab^
	Hexadecatrienoic acid (C16:3)	0.11 ± 0.00 ^b^	0.14 ± 0.00 ^a^	0.16 ± 0.00 ^a^	0.16 ± 0.01 ^a^
	Octadecenoic acid (18:2)	18.93 ± 0.56 ^b^	24.45 ± 0.77 ^a^	24.37 ± 0.93 ^a^	24.44 ± 1.12 ^a^
	Octadecatrienoic acid (C18:3)	44.37 ± 1.34 ^c^	56.66 ± 4.07 ^c^	61.43 ± 8.82 ^b^	71.18 ± 3.61 ^a^
	Total fatty acids	277.6 ± 5.14 ^c^	330.8 ± 12.0 ^b^	419.7 ± 5.4 ^a^	451.2 ± 12.2 ^a^
	Saturated fatty acids	24.1 ± 0.21 ^b^	25.67 ± 0.35 ^ab^	30.19 ± 0.21 ^a^	26.1 ± 0.37 ^ab^
	Monounsaturated fatty acids	0.92 ± 0.01 ^b^	1.06 ± 0.02 ^b^	1.28 ± 0.04 ^ab^	1.34 ± 0.03 ^a^
	Polyunsaturated fatty acids	35.99 ± 0.97 ^b^	46.07 ± 1.38 ^a^	47.00 ± 1.46 ^a^	46.97 ± 1.86 ^a^
	Total lipide	139.0 ± 12.8 ^b^	175.5 ± 16.9 ^b^	210.7 ± 20.5 ^a^	234.4 ± 7.0 ^a^
	Total protein	131.1 ± 12.9 ^b^	261.1 ± 25.1 ^a^	137.0 ± 22.7 ^b^	236.7 ± 9.2 ^a^
	Total Sugar	199.7 ± 27.8 ^b^	317.0 ± 30.5 ^a^	328.2 ± 39.8 ^a^	470.1 ± 43.1 ^a^
	Crude fiber	3.7 ± 0.44 ^a^	2.5 ± 0.24 ^b^	3.1 ± 0.27 ^a^	2.9 ± 0.53 ^b^

The superscript uppercase letters (a–d) indicate statistically significant differences between the samples. The statistical significance of the relative abundances was determined by Tukey’s post-hoc test, with *p* ≤ 0.05.

**Table 3:** The effect of laser light treatment (5 nm at 632 nm), BAP priming (25 µM for 8 h), and laser combined with BAP treatment on secondary metabolites of *Linum usitatissimum* sprouts.

Mg·g^−1^ FW	Control	Laser	BAP	Laser + BAP
Steroid	117.0 ± 11.07 ^b^	114.6 ± 11.04 ^b^	172.8 ± 12.83 ^a^	190.2 ± 7.53 ^a^
Tannin	52.7 ± 3.63 ^b^	58.4 ± 5.63 ^b^	73.8 ± 5.40 ^a^	71.9 ± 3.44 ^a^
Saponin	11.3 ± 0.96 ^c^	13.7 ± 1.31 ^c^	16.8 ± 1.11 ^b^	19.8 ± 0.40 ^a^
Total alkaloid	25.8 ± 3.80 ^b^	26.3 ± 2.53 ^b^	34.6 ± 5.28 ^a^	45.8 ± 1.84 ^a^
Caffeic acid	4.18 ± 0.34 ^c^	5.37 ± 0.55 ^b^	2.55 ± 0.21 ^d^	6.01 ± 0.44 ^a^
Ferulic acid	0.05 ± 0.00 ^c^	0.08 ± 0.01 ^b^	0.32 ± 0.01 ^a^	0.10 ± 0.04 ^b^
Galic acid	5.18 ± 0.55 ^b^	6.57 ± 0.14 ^b^	6.93 ± 1.52 ^b^	8.48 ± 0.66 ^a^
p-Coumaric acid	1.59 ± 0.05 ^b^	1.70 ± 0.17 ^ab^	1.30 ± 0.12 ^b^	2.31 ± 0.34 ^a^
Chlorogenic acid	0.17 ± 0.01 ^a^	0.13 ± 0.01 ^a^	0.10 ± 0.01 ^b^	0.09 ± 0.02 ^b^
Total phenols (TPC)	12.6 ± 0.88 ^b^	9.4 ± 0.90 ^c^	12.9 ± 0.64 ^b^	20.2 ± 2.00 ^a^
Catechin	1.81 ± 0.06 ^a^	1.52 ± 0.15 ^a^	0.78 ± 0.13 ^b^	0.95 ± 0.02 ^b^
kaempferol	0.94 ± 0.09 ^b^	1.37 ± 0.14 ^a^	1.10 ± 0.24 ^b^	1.30 ± 0.21 ^a^
Quercetin	2.33 ± 0.11 ^b^	3.57 ± 0.36 ^a^	1.11 ± 0.10 ^c^	2.25 ± 0.06 ^b^
Luteolin	0.08 ± 0.00 ^b^	0.12 ± 0.01 ^a^	0.04 ± 0.00 ^c^	0.10 ± 0.02 ^ab^
Apigenin	0.24 ± 0.01 ^ab^	0.37 ± 0.04 ^a^	0.12 ± 0.01 ^b^	0.23 ± 0.01 ^a^
Naringenin	1.72 ± 0.06 ^a^	2.23 ± 0.23 ^a^	0.69 ± 0.03 ^b^	1.75 ± 0.07 ^a^
Rutin	1.63 ± 0.07 ^a^	1.26 ± 0.13 ^ab^	0.78 ± 0.07 ^b^	1.23 ± 0.14 ^ab^
Velutin	0.01 ± 0.00 ^ab^	0.02 ± 0.00 ^a^	0.00 ± 0.00 ^b^	0.02 ± 0.00 ^a^
Tricin	1.39 ± 0.06 ^a^	1.72 ± 0.18 ^a^	0.52 ± 0.07 ^b^	1.42 ± 0.05 ^a^
vitexin	0.68 ± 0.03 ^b^	1.01 ± 0.10 ^a^	0.46 ± 0.04 ^b^	0.73 ± 0.04 ^ab^
Total flavonoids (TFs)	0.3 ± 0.02 ^b^	0.3 ± 0.03 ^b^	0.3 ± 0.01 ^b^	0.8 ± 0.05 ^a^

The superscript uppercase letters (a–d) indicate statistically significant differences between the samples. The statistical significance of the relative abundances was determined by Tukey’s post-hoc test, with *p* ≤ 0.05.

**Table 4:** The ash and mineral response of *Linum usitatissimum* sprouts to laser light treatment (5 mn at 632 nm), BAP priming (25 µM for 8 h), and laser treatment combined with BAP.

mg/g^−1^ DW	Control	Laser	BAP	Laser + BAP
Ash	1.6 ± 0.47 ^b^	2.3 ± 0.22 ^a^	1.9 ± 0.49 ^b^	3.2 ± 0.25 ^a^
N	28.95 ± 4.09 ^b^	40.90 ± 6.62 ^b^	46.02 ± 8.45 ^ab^	49.79 ± 6.55 ^a^
P	5.75 ± 0.85 ^b^	5.80 ± 0.03 ^b^	5.74 ± 1.58 ^b^	7.48 ± 0.26 ^a^
K	11.98 ± 0.86 ^c^	16.31 ± 1.47 ^b^	12.39 ± 1.82 ^c^	21.73 ± 1.80 ^a^
Ca	1.78 ± 0.50 ^d^	4.00 ± 0.93 ^a^	2.55 ± 0.54 ^c^	3.41 ± 0.71 ^b^
Mg	0.70 ± 0.27 ^b^	2.99 ± 0.76 ^a^	2.01 ± 0.88 ^a^	2.67 ± 0.85 ^a^
Na	0.37 ± 0.07 ^b^	0.25 ± 0.27 ^b^	0.79 ± 0.26 ^a^	0.49 ± 0.21 ^ab^
Fe	0.16 ± 0.01 ^c^	0.23 ± 0.05 ^b^	0.20 ± 0.02 ^b^	0.28 ± 0.06 ^a^
Zn	0.04 ± 0.00 ^b^	0.04 ± 0.01 ^b^	0.06 ± 0.01 ^a^	0.05 ± 0.01 ^ab^
Cu	0.08 ± 0.01 ^d^	0.19 ± 0.01 ^b^	0.12 ± 0.02 ^c^	0.23 ± 0.04 ^a^

The superscript uppercase letters (a–d) indicate statistically significant differences between the samples. The statistical significance of the relative abundances was determined by Tukey’s post-hoc test, with *p* ≤ 0.05.

**Table 5:** The antimicrobial activity of *Linum usitatissimum* sprouts treated with laser light, BAP, or laser treatment combined with BAP.

		Control	Laser	BAP	Laser + BAP
		Diameter of Inhibition Zones (mm)			
Gram-positive	*Staphylococcus saprophyticus*	13.1 ± 0.7 ^c^	21.8 ± 2.53 ^a^	19.4 ± 2.8 ^b^	20.6 ± 1.8 ^a^
	*Staphylococcus epidermidis*	8.9 ± 0.54 ^c^	19.22 ± 1.96 ^a^	15.1 ± 4.4 ^b^	16.4 ± 1.20 ^b^
	*Enterococcus faecalis*	16.6 ± 1.6 ^a^	16.26 ± 0.79 ^a^	11.0 ± 3.1 ^b^	16.6 ± 1.51 ^a^
	*Streptococcus salivarius*	6.9 ± 0.39 ^b^	14.26 ± 2.89 ^a^	13.0 ± 5.0 ^a^	12.9 ± 2.7 ^ab^
Gram-negative	*Escherichia coli*	6.1 ± 0.39 ^b^	10.31 ± 11.03 ^a^	6.17 ± 0.9 ^b^	8.5 ± 3.81 ^a^
	*Salmonella* *enterica* *Typhimurium*	9.3 ± 0.9 ^c^	19.95 ± 4.42 ^a^	13.2 ± 3.7 ^b^	16.1 ± 2.9 ^ab^
	*Pseudomonas aeruginosa*	19.9 ± 1.1 ^c^	26.6 ± 2.04 ^a^	22.8 ± 3.3 ^b^	26.2 ± 1.5 ^a^
	*Proteus vulgaris*	21.3 ± 4.0 ^a^	18.8 ± 2.5 ^b^	17.0 ± 0.3 ^b^	21.6 ± 0.6 ^a^
	*Enterobacter aerogenes*	15.9 ± 1.0 ^a^	14.53 ± 0.93 ^b^	14.07 ± 1.0 ^b^	16.8 ± 0.4 ^a^
	*Serratia marcescens*	6.4 ± 0.78 ^b^	8.21 ± 1.39 ^a^	6.04 ± 0.03 ^b^	7.84 ± 0. ^ab^
	*Salmonella typhimurium*	14.7 ± 1.4 ^b^	14.00 ± 2.34 ^b^	17.16 ± 0.1 ^a^	17.3 ± 1.3 ^a^
Fungus	*Candida albicans*	12.1 ± 2.9 ^a^	11.65 ± 1.82 ^ab^	9.58 ± 1.4 ^b^	12.65 ± 0.9 ^a^
	*Candida glabrata*	3.6 ± 0.4 ^b^	8.19 ± 2.03 ^a^	5.10 ± 1.5 ^b^	6.43 ± 1.2 ^ab^
	*Aspergillus flavus*	11.2 ± 1.7 ^b^	17.0 ± 1.96 ^a^	15.0 ± 2.2 ^ab^	16.4 ± 1.27 ^a^

The superscript uppercase letters (a–d) indicate statistically significant differences between the samples. The statistical significance of the relative abundances was determined by Tukey’s post-hoc test, with *p* ≤ 0.05.

## Data Availability

Not applicable.
